# On Prostitution in the City of Paris, &c.

**Published:** 1837-07

**Authors:** 


### Art. IV.

*De la Prostitution dans la Ville de Paris, considérée sous le Rapport de l'Hygiène publique, de la Morale et de l'Administration; ouvrage appuyé de Documens statistiques puisés dans les Archives de la Préfecture de Police; avec Cartes et Tableaux.* Par A. J. B. Parent-Duchatelet, &c. *Précédé d'une Notice historique sur la Vie et les Ouvrages de l'Auteur, par* Fr. Leuret.—*Paris,* 1836. 8vo.

*On Prostitution in the City of Paris, considered in Relation to Public Hygiène, Morals, and Police; founded on Official Documents; with Plans and Tables.* By A. J. B. Parent-Duchatelet, &c. *With a Biographical Notice of the Author, by* Fr. Leuret.—*Paris,* 1836. Two vols. 8vo. pp. 624 and 580.

This work is the production of a very remarkable person, who attached himself with singular zeal to the investigation of the effects upon society of many moral and many physical nuisances. In the book before us, the very title of which is calculated to alarm the general reader, the author lifts up the veil which usually conceals from the well-regulated portion of society the mode of life of the abandoned and the profligate, and discloses scenes of vice, and of concomitant wretchedness, painfully instructive to all, and from the contemplation of which the philanthropist, and especially the medical philanthropist, should not affectedly turn away. He withdraws us for awhile from the ordinary ways of men, from the honest light of day, from the pursuits which stimulate honorable competition, and from the innocent gaiety which dreads no public or private censure, to the darkest retreats of infamy, to the dreadful walks of unholy industry, to dens of vicious gratifications, which would fain shun the eye of God and man. He takes from before us the accustomed images of female loveliness and appropriate virtue, and the charm of unblemished womanhood, and shews us horrid visions of dissoluteness, and the ruin of young and innocent hearts, and the fierce abandonment or strong depravity which alike banish every womanly attribute. But, with these pictures, he also shews us the care of an enlightened government, unceasingly employed to lessen all the vices and miseries incidental to great cities, and the never-wearied labour of many excellent persons who seek to reclaim the unfortunate, and bring back the depraved to habits of virtue.

The "Annales d'Hygiène publique" contain many of the results of the previous labours of M. Parent-Duchatelet, whose whole life appears to have been devoted, with the ardour of an enthusiast, and with an industry that no difficulties could discourage, to the amelioration of the physical and moral condition of his countrymen. M. Leuret has prefixed to the work before us a highly interesting account of these great services, the perusal of which, and of the affecting testimony therein given to the private virtues of his character, have heightened our regret for his loss, at the early age of forty-five. The work before us, like all his other productions, is the result of a long series of most accurate enquiries, pursued, in this instance for eight years, amidst circumstances which would have disgusted or affrighted a man of less resolution and humanity. We shall endeavour to lay such portions of its contents before our readers as may be in any degree capable of useful application. The statements contained in the work are founded on facts sought out with infinite research in the public offices, especially the *Bureau des Mœurs*, and from public functionaries, physicians, surgeons, students, and nurses of hospitals, the keepers of prisons, and the humane visiters of these sad abodes; and from the inhabitants of brothels, which for this purpose the author visited both by day and night, always accompanied by a medical officer or a peace officer. Nor was he content, as observers too commonly are, to note down the circumstances observed as happening *sometimes*, or *often*, or *very often*, but he spared no pains to reduce them to figures by exact numerical calculation.

The first result of this exactness is to correct popular errors. For seventy years past the city of Paris has been thought to contain about 20,000 prostitutes; whereas, the actual number is not quite 4,000 at the present time, and the number twenty years ago was not 1,300. It is probable that a similar error has distinguished "London, with her 100,000 prostitutes;" the real number appearing not to be more than one-tenth of that amount; although the London calculations are more vague than those of Paris, in consequence of the system of registration in the French capital.

Instead of loose accusations of Paris or the provinces, as regards the number of depraved women proceeding from different parts of France, M. Parent-Duchatelet gives an exact account of the proportion of prostitutes furnished to Paris from all the departments of France, and from foreign countries, since the year 1816; and he accompanies this account with a map of France, in which the districts from whence the greatest number of common women have proceeded are shaded more darkly than the rest. Those north and south of the capital, touching the department of the Seine, are of the deepest hue, as might be expected; and the northern departments are, generally, of a darker shade than the rest. Perhaps, little is gained by this representation more than a confirmation of a very probable circumstance,—namely, that in the vicinity of the larger cities or towns, and especially of sea-ports, the number of prostitutes is always the greatest. Thus, the darkest spots remote from Paris in the map are the neighbourhoods of Marseilles, Lyons, and Bourdeaux, places wherein wealth most abounds, and where the population is considerable, and the communication with the capital easy and frequent.

A more interesting enquiry is that which regards the condition in life from which the greatest number of prostitutes spring; for this may throw light on the causes of the depravity of manners, and direct the legislator to means for its control. Such an enquiry is evidently difficult, on account of the numerous deceptions incidental to it; but much of this difficulty was obviated by a decree, enjoining that, to the registration of each prostitute, should be added a register of the condition of the parents, attested by two witnesses; the second witness, however, being generally no other than one of the mayor's officers, those functionaries acquiring, apparently, a facility in making attestations by dint of witnessing them in their daily round of duties. It would appear that every step of the author's enquiry was destined to overthrow some popular theory. Imagination had invested many of the public women of Paris with high birth and connexions; whereas, out of 828 registrations, only four had any pretensions to rank, and the rest were nearly all from the different classes of artisans. Out of 2,500 provincial registrations, the results were the same. That poverty and ignorance prevail in the unfortunate families of which so many daughters desert the paths of virtue, is proved by the fact that one-third of the fathers were unable to write their names, and this in Paris, where primary instruction is almost universal: in the departments the proportion of those unable to write was still greater. One-fourth of the prostitutes of Paris were found to be of illegitimate birth, of whom only one-half were acknowledged by their fathers; a fact which strongly exhibits the train of unhappy consequences dependent on illicit intercourse. Of 3,084, whose occupations previous to their entering on a course of prostitution is mentioned, only three possessed property, and the richest had 1000 francs (40*l.* sterling) per annum: the rest were chiefly workwomen of various kinds, and servants, and the greater number were employed in sedentary occupations, in workshops, poorly paid, and liable to be deprived of work by fluctuations in trade and fashion. They were also, for the most part, without education; 2,332 out of 4,470 were unable to write; and consequently they had few resources against absolute want. A curious table at p. 86 indicates the ages at which 3,248 inscribed themselves as common prostitutes. We observe that there were 2 at ten years, 3 at eleven, 3 at twelve, 6 at thirteen, 20 at fourteen, and 51 at fifteen. The number then increases to above 100 at each age; at twenty, it is 389; it falls at twenty-eight years to 101, and at twenty-nine to 57; goes on decreasing until at forty there are only 9, and at fifty only 4. A few inscriptions occur after the age of fifty, but they are chiefly of persons imported from the departments, who have probably been of loose life for many years.

Although it is highly probable that many prostitutes become such in consequence of a love of idleness, and pleasure, and fine clothing, a great number are driven to a life for which they have no inclination by the extremity of misery. Many are brought from the country by their seducers, and, being abandoned in Paris, and without resources, are an easy prey to the procuresses, who are ever on the watch for them. Many also, it would seem, go upon the town to escape the unkind or unwelcome discipline of their parents. Hospitals, and houses for servants out of place, afford opportunities of which the older bawds dexterously avail themselves. Very often an ill-managed household causes young women to abandon their homes, where they have been already accustomed to bad examples. Some there are, also, who expose themselves to prostitution in order to support their children or their parents; and some few who, in following the same wretched life, appear only to obey the depravity of their physical temperament.

M. Parent-Duchatelet seems very unsatisfactorily to dispose of the question of prostitution depending on a high state of civilization. The truth is, that in all states of mankind, savage and civil, there is a strong tendency to this error in morals; and it is probable that the remedy will not be found until communities are so happily ordered that early marriages cease to be looked upon as crimes, and children as misfortunes. The nearly equal proportion of male and female births would seem intended to provide for the marriage of many who, in all forms of society, pine in singleness; but no means have yet been discovered for ensuring an amount of individual prosperity which gives encouragement to the union of all nubile persons; and, so long as this continues, many women must fade unmarried, and many men and many women will continue to seek gratifications that entail less ruin upon them than marriage threatens.

M. Parent-Duchatelet devotes the second chapter of his work to the *Manners and Habits of Prostitutes*, which, as might be expected, present a singular mixture of the effects of evil associations with the results of early impressions and original character. Amidst shameless vices, some flowers of virtue still grow wild. The base stamp has not wholly superseded the first divine impression; and, in the wreck of many female attributes, there are surviving affections which show that the heart cannot become all evil. But these redeeming features seldom become known to those likely to reflect much upon them; they make a momentary impression upon the libertine, but no more. It is the philanthropist, who visits these victims of vice in hospitals and in prisons, in sickness and in sorrow, who becomes fully acquainted with the depths of the heart wherein lie thoughts which have seldom before been uttered. The unfortunate women also entertain, it seems, the keenest sense of their lost and abandoned condition, and are often only to be roused from the extremest despondency by the hope of being once more restored to honest life. The greater number of them have had no religious education: early sold to vice by vicious parents, even the idea of a God has not been instilled into the minds of many of them. They are disposed to fanaticism, and seldom inscribe themselves, or go to the dispensaries for examination, on a Friday. Notwithstanding their general abandonment of manners, all womanly modesty is not yet banished, even in the hospitals for the sick, where it is perhaps put to the severest proof; and the public indecencies which characterized the period of the first revolution, but which were not unknown before that time, are witnessed no longer. As few of them have received any education, their irregular lives produce the most desultory mental habits; and noise, variety, and excitement seem to become essential to their existence. In prison, in the hospitals, or in the penitentiaries, "it is impossible," says M. Parent-Duchatelet, "to describe the extent of their loquacity:" but of late years their conduct in these situations has become more orderly.

Among the customs traceable to a kind of sentiment, those of them who consort with the soldiers adopt the habit of having figures or initials marked in blue upon their shoulders or under their breasts, with devices emblematical of constancy. These marks, as might be expected, often become undoubted proofs of the weakness of such constancy; twenty or thirty vows of "love till death" being sometimes found thus inscribed on the body of the same individual. The older prostitutes have often inscribed upon themselves the names of women for whom they entertain a friendship, and in these cases alone the seat of the inscription is between the pubes and the umbilicus.

The intervals not employed in their peculiar avocation are generally passed in idleness; sometimes, however, in reading; and it is remarkable that the books found among them are never of that immoral class for which the Parisian capital enjoys an unenviable celebrity. The Parisian prostitutes are described as negligent of personal cleanliness, an attention to which, in this country, often gives advantages to loose women above those of better character. They are also accustomed both to eat and drink to excess; and a disregard to truth, and a proneness to anger, are enumerated among their faults. On the other hand, they are charitable and generous to excess, often giving clothes and money to those of their own class, from whom they expect nothing but ingratitude, and sometimes making daily donations of bread to individuals or families more wretched than themselves. They rarely betray one another. Those of them who become mothers perform all their maternal duties with great solicitude, and look upon their new position as an advance towards a more decent kind of life; and in these circumstances both the mother and infant are overwhelmed with the kind attentions of her unfortunate companions. Contemplate human beings when and where we may, we see the same feelings struggling through the accidents which surround them. Notwithstanding the obstacles that may readily be imagined to be in the way of those virtuous friendships from which the human heart seeks its best consolations, the strong desire of attaching themselves to some one in particular, united with the wish for protection, often unites prostitutes to men of ferocious character, whom they supply with the means of living, and whose ill-treatment they endure with a patience amounting to infatuation. These men lead idle and abandoned lives, without any feeling of shame, deriving from the poor creatures who are attached to them the money which they lose at the gaming-table, or spend in sensual gratifications. There is nothing very peculiar in these circumstances, of which notorious examples might be adduced even from the ranks of the aristocracy of our own country. The older prostitutes would seem to be adepts in the art of gaining the affections of younger women, between whom and themselves unnatural attachments are asserted not unfrequently to exist, attended even with more than ordinary jealousy: among those who are depraved to this extent, the most unpardonable offence is the transference of the affections of either party to a man.

Our insular ignorance makes us somewhat at a loss to follow the divisions adopted in the chapter wherein are described the different ranks and classes of the prostitutes of Paris. The *femmes galantes* seem to be kept mistresses; but the *femmes à parties*, whose manners and mental accomplishments add to the seductions of the gaming-houses, are unknown in our manners. The *femmes de spectacles et de théâtres*, also, appear to be a class distinct from those known among us, and whom there could be no difficulty in enumerating among prostitutes; whereas, in Paris, they escape the vigilance of the police no less than the two higher classes just mentioned: "mulier quæ non palam, sed passim et paucis, sui copiam facit, actio competit adversus eum qui eum meretricem vocavit;" a very nice distinction. The numerous other divisions dwelt upon by M. Parent-Duchatelet require little notice: they are as various as the different classes of society to which they offer opportunities of vice. An ascent or descent from one rank to another seems an uncommon event: each prostitute takes her place at once, and keeps it, although all aspire to the dignity of *dame de maison;* in other words, to being some day at the head of an establishment of their own.

The physiological peculiarities of prostitutes are not very remarkable, and they are as easy, we think, to be accounted for as the psychological characters which have been already mentioned. Their general embonpoint after a certain age arises naturally enough out of good eating, drinking, and indolence, without any necessity of ascribing it to the use of mercury for the cure of disease. The change of their voices is, we believe, chiefly observable in those who drink and are exposed much to the night-air. It is akin to the "gin-voice" so well known in London.

In the section on the colour of the hair and eyes of prostitutes, we find nothing worthy of comment. That on the sexual organs contains some important facts, hitherto unknown to medical jurists. It commences with an instructive anecdote. Two young women were grossly insulted in the street, and proclaimed to be prostitutes. The offence became the ground of public complaint; and the young women, asserting that they were virgins, offered to submit to a medical examination. The examining officer, a man of experience and discretion, pronounced it impossible to give a decided opinion concerning one of them, but he suspected that the other was not a virgin. Some time afterward it was proved that both of the young women were common prostitutes at the time of the examination, and had been repeatedly affected with syphilis. M. Parent Duchatelet had repeated opportunities of examining young women after forcible defloration, and he generally found that the details given to him by them (they were chiefly children,) gave him more information than the state of the sexual organs. He observes, that no opinion is more generally received than that the sexual organs of prostitutes *must* present peculiar alterations; and yet the most careful attention to this subject, on the part of the physicians and surgeons of the dispensary, and police hospital, and prison infirmaries, has proved that "the genital organs of prostitutes do not present any special alteration or any change peculiar to them: in this respect, there exists no difference between them and the most respectable married woman." (P. 214.) The frequent employment of the speculum has also proved that the width or narrowness of the vagina vary in different individuals, without reference to the length of time during which they have been accustomed to indiscriminate sexual intercourse. Young prostitutes, who have never been pregnant, have not unfrequently a large vagina, and old prostitutes sometimes retain what is erroneously considered the characteristic smallness of virginity. These are facts of the highest importance, and should be well remembered by medical witnesses.

The test of pregnancy, mentioned on the authority of M. Jacquemin, namely, a violet or deeper colour of the vaginal mucous membrane, would admit, one would suppose, of easy verification: its value in cases of suspected, doubtful, and simulated pregnancy, is evident.

Considerable difficulty seems to have been met with by M. Parent-Duchatelet in investigating the general condition of prostitutes as respects the function of menstruation. In general, however, it would appear that this function is seriously interrupted, and often for a great length of time. Of those who enter the Penitentiary, all are in the state of amenorrhœa suppressionis, and they remain so for a considerable period. This irregularity is accounted for by the various imprudent practices and irregularities of the lives of prostitutes; and in some instances, perhaps, by the artificial means had recourse to for the express purpose of preventing frequent periodical interruptions of their mode of obtaining a livelihood.

These facts are accordant with the general, and, we should say, the correct opinion of the infecundity of prostitutes; the exceptions to which we deem M. Parent-Duchatelet to have raised into too much importance. These exceptions are very rare; and the principal midwife of the Maternity Hospital, where the delivery of prostitutes who have become pregnant generally takes place, reports that their labours are usually difficult, that it is commonly necessary to employ the forceps, and that the infants seldom survive. Many, however, are said to become impregnated, and to have abortions at an early period. They generally assert that they know by whom they have become impregnated, and the person named is usually some one to whom they are attached. The mortality among their unhappy children is excessive: of eight born in prison, four die within the first fortnight, and the other four within the first year; of ten infants born in the hospital in one year, five died almost as soon as born, and the other five before the complete recovery of the mother.

The most frequent maladies to which prostitutes are subject are syphilis and the itch. They are also subject to uterine hemorrhages; such being frequent in the hospital of the prison in which they are received, and often occurring at the age of fourteen or fifteen, at which age they are unusual in other women. Not unfrequently they are affected with tumours of the labia majora, on one side, enlarging at the menstrual periods, and sometimes attaining a considerable size. They are indolent, rarely fibrous, and ordinarily contain a thick albuminous fluid, extraordinarily fetid. Abscesses of the labia are of frequent occurrence, and heal without serious consequences: those which form in the recto-vaginal partition are more troublesome, frequently degenerating into troublesome fistulæ: these are often observed to be co-existent with a hard engorgement of the labia; and their conjunction with phthisis is remarked in numerous instances, (*presque toujours.*) It is a commonly received opinion in all countries, that prostitutes are particularly obnoxious to cancer of the uterus; but, according to M. Parent-Duchatelet, this notion is not well founded, nor are prostitutes peculiarly liable to elongations, irritation, or inflammation of the neck of the uterus. The frequent occurrence of uterine cancer in unmarried women, examples of which were formerly very numerous in religious establishments, throws considerable doubt on the theory of such supposed occurrences in prostitutes.

Prostitutes do not seem to be peculiarly liable to hysterical or spasmodic attacks. They were at one time frequent in the Venereal Hospital, but M. Cullerier cured them, or rather prevented them, by the threat of plunging the patients so attacked into cold water, and branding them with hot irons. His success was equal to that of Boerhaave. Before this was threatened, every patient had fits after being a few weeks in the hospital, and they called them "coming out of their mercury," (*revenir de son mercure.*) Those who wholly abandon their vicious habits, and go to the Penitentiary, become subject for a year or two to hysterical seizures and cerebral congestion, disturbing in some degree the intellectual functions. Mental imbecility is frequently observed in prostitutes somewhat advanced in life. Mental derangement is not uncommon. Of 105 received into the Salpêtrière, from 1811 to 1815, the youngest was sixteen years of age, and the oldest sixty-two:

| | | | |
|---|---|---|---|
| Between | fifteen and twenty years of age, there were | | 4 |
| — | twenty and twenty-five, | — | 15 |
| — | twenty-five and thirty, | — | 26 |
| — | thirty and thirty-five, | — | 25 |
| — | thirty-five and forty, | — | 18 |
| — | forty and forty-five, | — | 10 |
| — | forty-five and fifty, | — | 5 |
| — | fifty and fifty-five, | — | 0 |
| — | fifty-five and sixty, | — | 1 |
| — | sixty and sixty-five, | — | 1 |
| | | | 105 |

The causes of the insanity were,

| | |
|---|---|
| Unknown, in . . . | 37 |
| Fear, . . . | 3 |
| Licentious excesses, . . | 3 |
| After delivery, . . . | 8 |
| Excessive misery, . . | 11 |
| Mercurial treatment, . . | 3 |
| Abuse of wine, . . . | 13 |
| Mental vexations, (*chagrins profonds,*) | 27 |
| | 105 |

Among the 27 last mentioned, the vexation arose in 14 from the desertion of their lovers; in one it was occasioned by being recognized as a prostitute by one of her own countrymen; in another it was produced by her being for the third time delivered of a dead child. The form taken by the malady was,

| | |
|---|---|
| Melancholy, in . | 36 |
| Mania, . | 43 |
| Dementia, . . | 18 |
| Not distinctly defined, | 8 |
| | 105 |

It is a curious fact in the annals of prostitution, that bodily infirmities sometimes seem to be recommendations. Lame women, whose legs are defective in length or deformed, and others still more lamentably afflicted, have been known to be in particular request. Pregnancy is well known to enhance their value. Among the common women of Paris, there was one who was deaf and dumb.

Scrofula, in all its forms, is extremely prevalent among the Parisian prostitutes, and greatly aggravates the evils of syphilis; but their general health does not appear to be worse than that of other women. They even suffer less than many married women, and than those employed in sedentary occupations; and they recover quite as well from attacks of ordinary illness.

There are considerable portions of M. Parent-Duchatelet's work which we feel ourselves compelled to pass over without remark, although containing much curious and valuable information, because such information chiefly concerns the general reader. We have already referred more than once to the public registry of the prostitutes of Paris, a plan suggested so long ago as 1765, and again in 1771, for the protection of public morals, and for the superintendence of the health of the prostitutes. After a period of anarchy, an imperfect plan of registration seems first to have been adopted in 1796, but it received great amendments in 1804, and was further improved in 1828. This system is too remarkable to be left out of our notice of the work before us. Of those inscribed on the register, some present themselves for that purpose, some are brought by the mistress of an establishment, and some by the public inspectors. The woman's name, age, place of birth, occupation, and residence are enquired into. She is asked whether she is married or single, or a widow; if her father and mother are living, and how they are occupied; whether she lives with them, or when and why she left them; if she has had or has any children; how long she has lived in Paris; whether any one in Paris has authority to claim her; whether she has ever been arrested by the police, how often, and wherefore; if she has already lived the life of a prostitute, and how long; if she has ever had or has any venereal affection; if she has received any education; what motives determine her to register herself; with many other questions suggested by her answers, from which the practised interrogators obtain a full knowledge of her character and habits, and are at no loss how to class her. She is then visited and examined by medical men appointed to that duty, who report her state of health. Various other measures are taken to ascertain the truth of the depositions; such as writing to the mayor of the alleged town of birth, &c. Those who are diseased are sent to the hospital; the hardened and corrupt are punished, and every effort is made to reclaim the young, the comparatively innocent, or those who come to be inscribed in a fit of anger or despair; and they are often sent to their families. Once in two or three years an instance occurs in which the woman offering herself for inscription declares herself, and is declared by the medical examiners, to be a virgin: in this case the inscription is refused. The greater number, however, are women of loose life and diseased. In a thousand inscriptions, about 600 are inscribed of their own accord, and 350 by the mistresses of establishments.

The mistresses of establishments (*dames de maison,*) are persons who affect considerable consequence, and exercise in their establishments despotic sway. Their letters of application for licence abound in high-flown language, and they often assign noble, honorable, and even religious motives for desiring permission to open a house of ill-fame. Some lay much stress on their own excellent character and grave manners, some on their regard for the health of the public, and some on the duty of making a provision for their children. M. Parent-Duchatelet inserts several of these applications, and among them one from a venerable lady of eighty-two, who asks leave to keep her daughter and granddaughter in a state of genteel prostitution on account of her advanced time of life, and because she feels that she must soon "appear before her Creator," and therefore wishes to provide her children with the means of existence. A curious map is given, indicative of the quarters of Paris in which prostitutes most abound, and which shows how much they congregate where trade and wealth are greatest.

Referring the reader for fuller particulars on these and many other subjects connected with the section of society which occupies the author's attention, to the two thick volumes themselves, we believe a stronger interest will be felt in that part of it which relates to the sanitary surveillance of the health of prostitutes, which is one of the great objects of the system of registration, for the common preservation of society. Casuists may certainly maintain the impropriety of interfering between vice and its natural punishment, but the interests of communities as much demand that medicine should step in between libertinism and consequent suffering, as between idleness and luxury and their consequences. In either case, to withhold aid, would be to involve the innocent as well as the guilty in countless miseries. It is justly observed by the author, that no pestilence ever committed more havoc than syphilis; of which the effects are most frequently developed in the young and strong, and transmitted by them to a tainted and frail and innocent progeny. Nor is the dread of such evils salutary, or in any material degree prohibitory of illicit pleasures. Paris, with its registers and sanitary regulations, is not more vicious than London or any other large capital; and, at the best, the fear inspired by disease can only operate to prevent intercourse with common women, the least widely hurtful to society of all modes of unlawful licentiousness, since it corrupts morality the least, and the least disseminates wretchedness throughout virtuous families.

Although the question of sanitary regulations had frequently occupied the attention of the French government, they were only fully organized about the commencement of the present century, when a provision was also made for the expenses attendant on them by a tax of three francs (two shillings and sixpence,) a month on each separate or isolated prostitute, and twelve francs on each *dame de maison*. The collection of this tax was intrusted to two surgeons, who were appointed to the duty of making periodical visits and examinations, and who for a time contrived to exact a large annual sum from the wealthy prostitutes, and entirely neglected the poorer; and, although they established the dispensary, they shamefully neglected the duties it imposed upon them, and especially that of making proper reports; but still contrived to make about 1200*l*. a year by the institution. In 1810 the dispensary was wholly reformed; and it was then ordered that every prostitute should be visited twice in each month; that a regular note of these visits should be sent to the prefecture; that the tax should be collected by a proper officer, and the surplus, after payment of the salaries of the medical men, should be applied to the improvement of the institution. The first physician and first surgeon were to have each a salary of 6000 francs (240*l*.); the second physician and surgeon, each 3000 francs: two students, one of medicine and one of surgery, each 1800 francs; and the apothecary, who also was to keep the registers, 2400 francs. M. Pasquier, then at the head of the police, and to whom belongs the honour of improving this establishment, required weekly reports from the dispensary, and instituted a permanent commission, which was to meet monthly, and to superintend the working of the whole plan. During the four years in which M. Pasquier remained in office, the dispensary continued to be so well regulated that the number and severity of the venereal cases in Paris was sensibly diminished. The invasion of the Allies in 1814, and again in 1815, neutralized these good effects; but M. Anglès, who had succeeded M. Pasquier, met the evil by increased vigilance, causing the commissioners to meet twice in a month, attending their meetings himself, and increasing the frequency of the visits of the medical officers to one every ten days.

English medical writers and students, who resort for a time to Paris, have seldom an opportunity of witnessing the mode of inspection adopted by the official medical examiners, whose duties are partly performed at the dispensary, partly in the private establishments, and partly at the depôt of the prefecture of police. All the free or isolated prostitutes are bound to appear at the dispensary, for examination, twice in a month; those also are examined there who are inscribed for the first time, and those who are going into or quitting the establishments of the *dames de maison;* and also those who are retiring from prostitution, those who are going into the country, those who have been in prison, or in the hospital, or in temporary concealment. In the prison and at the hospital, the inspection is made in a private room, on a kind of table or bed, raised to the height of a *mètre*, resembling the table employed in the operation for the stone, with the addition of a small board in front, for the feet, and steps at the side. Many advantages are considered to arise from the adoption of this kind of table, which facilitates the employment of the speculum when necessary, and permits an inspection of the state of the anus and groins; the sensibility of the latter especially being often found indicative of uterine and other irritations. At the dispensary, however, this table cannot be employed, as the bonnets of the better-dressed prostitutes would be spoiled by the supine position; and they are therefore indulged with a high sofa, with a low reclining back, to which they ascend by several small steps, purposely devised to put the power of stepping up without pain to trial. It is calculated that each examiner may examine and register twenty-five patients in an hour; but, as the subjects to be examined come at all hours, and generally with repugnance, there are always two or three supernumerary medical officers in waiting, to prevent delay. Each prostitute has a card, on which the date and result of such examination is inscribed; and these are also copied into a book.

Every house kept by a *dame de maison* is visited by an examiner once a week, at a fixed hour; and an exact register is kept of every particular. If any one of the prostitutes is found diseased, the fact is notified to the mistress of the house, who is liable to heavy penalties if she permits any one to have access to her; and the woman pronounced to be affected must appear that day at the dispensary, and be again examined, preparatory to immediate removal to the hospital. If they evade this appearance at the dispensary, they are sent for, and, when cured, are punished for disobedience.

The examination at the depôt of the prefecture was instituted, that the opportunity might be thus taken of submitting loose women to examination who had long avoided it. The depôt is a kind of watch-house, or temporary prison for offenders taken up by the police.

Of this system the result has been, that, in twenty years, more than 20,000 women affected with syphilis have been promptly attended to, and prevented from spreading the disease. In the course of their experience, the inspectors have found that there are women who never become infected, whilst others are so constantly liable to infection that they almost pass their lives in the hospital. Of 250 received into the Venereal Hospital, eight had been on the town more than six years before becoming infected. The physicians of the dispensary are of opinion that infection is resisted by one-half of the public prostitutes. Women appear to be less liable to infection than men. All the medical examiners agree in stating that the dreadful cases of caries, &c., formerly so common, have become, and are progressively becoming more rare. As regards the influence of age, some of them think that buboes are only found in the young, and particularly those of a lymphatic temperament; rarely twice in the same individual, and less often in women than in men: they also consider vegetations as more common in very young women than in those who are more than twenty years of age. But other observers doubt the correctness of these distinctions. It is somewhat singular that, with all the exactness which characterizes the Parisian regulations, an attempt to ascertain the relative merits of the mercurial and non-mercurial methods of treating syphilis should have entirely failed.

Without extending our notice of M. Parent-Duchatelet's book far beyond the limits we are compelled to assign to it, we are unable to make any remarks on those not inconsiderable portions of his work which relate to the regulations of the police, and to the efforts made or making for the reformation of the unfortunate women to the illustration of whose life and condition he devoted so much time. The chapters relating to these subjects are of extreme interest to the general reader, and we have thought it proper to limit our analysis to matters more closely connected with the studies and duties of the medical practitioner, for whom the facts contained in this publication present much matter for useful reflection. As a specimen of industrious and careful observation, it is beyond all praise, and, with the exception of occasional diffuseness, there is nothing in it for the critic to object to. All the curious particulars which it comprehends are treated of with delicacy and judgment; and we can but lament that Paris was so soon deprived of so valuable a citizen, and our profession of so good and enlightened a man.

